# Oral selective estrogen receptor degraders in ER+/HER2− breast cancer: a systematic review with pooled analysis of metastatic randomized trials and narrative synthesis of adjuvant evidence

**DOI:** 10.3389/fonc.2026.1896847

**Published:** 2026-09-03

**Authors:** Bing Guan, Sijia Zhang, Rong Zhou, Yawen Chen, Lulu Li, Ping He

**Affiliations:** Department of Pathology, Suzhou BenQ Medical Center, Suzhou, China

**Keywords:** adjuvant endocrine therapy, breast cancer, camizestrant, elacestrant, ESR1 mutation, giredestrant, imlunestrant, oral SERD

## Abstract

**Background:**

Oral selective estrogen receptor degraders (SERDs) have become a key therapeutic option for hormone receptor-positive, HER2−negative advanced breast cancer. The recent presentation of lidERA—the first positive adjuvant Phase III trial of any oral SERD—necessitates a comprehensive evidence synthesis spanning both early and advanced disease settings, beyond the scope of existing meta-analyses confined to metastatic disease.

**Methods:**

This review was prospectively registered (PROSPERO CRD420261358420). We searched PubMed, Cochrane CENTRAL, and conference abstract archives (SABCS, ESMO, ASCO; 2020–2026) for Phase II/III randomized controlled trials comparing oral SERDs (giredestrant, imlunestrant, elacestrant, camizestrant, vepdegestrant, amcenestrant) with standard endocrine therapy in ER-positive, HER2−negative breast cancer. Progression-free survival hazard ratios in the metastatic setting were pooled using random-effects models. Prespecified sensitivity analyses included Bayesian re-analysis.

**Results:**

Ten randomized controlled trials met inclusion criteria (one adjuvant, n = 4,170; nine metastatic). Giredestrant significantly improved invasive disease-free survival in the adjuvant lidERA trial (HR 0.70; 95% CI 0.57–0.87; *P* = 0.0014). In the metastatic setting, pooled analysis of six oral degrader monotherapy trials (n = 2,502) showed a progression-free survival benefit (HR 0.815; 95% CI 0.718–0.924; *P* = 0.0014) with moderate heterogeneity (*I*² = 35.2%). The *ESR1*-mutated subgroup revealed a particularly robust and consistent effect (HR 0.617; 95% CI 0.527–0.723; *P* < 0.0001) with zero heterogeneity across five independent trials (*I*² = 0%). These findings were unchanged across all sensitivity analyses: restriction to Phase III trials only (HR 0.811; *I*² = 5.8%), exclusion of the PROTAC agent (HR 0.807), leave-one-out iteration (range 0.790–0.843), and Bayesian re-analysis (posterior median HR 0.814; 95% credible interval 0.678–0.969). Egger’s test did not detect significant funnel plot asymmetry (*P* = 0.40), though power was limited (k = 6).

**Conclusions:**

Oral SERDs confer a consistent progression-free survival benefit in advanced breast cancer, with the most robust and consistent effect in *ESR1*-mutated tumors. Giredestrant is the first oral SERD to achieve significantly improved invasive disease-free survival in the adjuvant setting, marking a potential new standard of care. This evidence synthesis across the full breast cancer continuum offers a framework for clinical decision-making as oral SERDs expand from advanced to early-stage disease.

**Systematic Review Registration:**

https://www.crd.york.ac.uk/prospero/display_record.php, identifier CRD420261358420.

## Highlights

- First systematic review of oral SERDs across adjuvant and metastatic breast cancer
- lidERA: first positive adjuvant Phase III trial of any oral SERD (HR 0.70, P = 0.0014)
- Metastatic pooled PFS HR 0.815 (95% CI 0.718-0.924, I²=35%), k=6 trials
- ESR1-mutated subgroup: HR 0.617, *I*²=0% across five independent trials
- Bayesian analysis and multiple sensitivity analyses validate reliability of all results

## 1 Introduction

Endocrine therapy is the cornerstone of systemic treatment for estrogen receptor-positive (ER+), HER2−negative breast cancer, which accounts for approximately 70% of all breast cancer diagnoses (1). In the adjuvant setting, tamoxifen and aromatase inhibitors have been standard of care for decades, reducing recurrence risk by 30–50% (2). Yet approximately 20% of patients with early-stage disease will develop recurrent disease within ten years despite completing adjuvant endocrine therapy (3). No novel endocrine agent has achieved superior adjuvant efficacy since aromatase inhibitors were introduced in the early 2000s.

In the advanced setting, cyclin-dependent kinase 4 and 6 (CDK4/6) inhibitors have transformed first-line treatment, but acquired resistance remains inevitable. Activating mutations in *ESR1*, the gene encoding estrogen receptor alpha, represent a major resistance mechanism—they drive ligand-independent receptor signaling and are detectable in circulating tumor DNA in 30–40% of patients with prior aromatase inhibitor exposure (4). Fulvestrant, the first clinically available selective estrogen receptor degrader (SERD), retains activity against *ESR1*-mutated tumors but is limited by intramuscular administration and suboptimal bioavailability. Oral SERDs—elacestrant, imlunestrant, giredestrant, camizestrant, and the subsequently discontinued amcenestrant—were designed to overcome these limitations through continuous oral dosing and improved target engagement (5).

Clinical evidence supporting this drug class has grown substantially in recent years. Elacestrant received FDA approval in 2023 for *ESR1*-mutated advanced breast cancer based on the EMERALD trial (6); imlunestrant received FDA approval in September 2025 based on the EMBER-3 trial (7). Two recent systematic reviews have synthesized these data in the metastatic disease setting (8, 9). However, the presentation of the lidERA Breast Cancer trial at SABCS 2025—the first positive Phase III adjuvant oral SERD trial, showing statistically significant improvement in invasive disease-free survival with giredestrant (10)—marks a pivotal development that existing evidence syntheses do not capture. Unlike prior reviews confined to advanced disease, the current study includes lidERA and evaluates oral SERDs across the full breast cancer continuum, from early-stage adjuvant through advanced/metastatic disease.

We performed a comprehensive systematic review and pooled analysis of Phase II/III randomized controlled trials evaluating oral SERDs across the breast cancer continuum. Our objectives were threefold: to provide an updated quantitative synthesis incorporating the most recent Phase III data; to present the first systematic synthesis of adjuvant oral SERD evidence; and to map the landscape of ongoing confirmatory adjuvant trials.

## 2 Methods

### 2.1 Protocol and registration

This review followed the PRISMA 2020 statement (11) and was prospectively registered in PROSPERO (CRD420261358420). An amendment authorizing inclusion of conference abstracts as grey literature was approved before data extraction (Amendment 1, May 2026).

### 2.2 Search strategy

We retrieved literature from PubMed and the Cochrane Central Register of Controlled Trials (CENTRAL) between January 2018 and April 2026. The Boolean search combined keywords for oral SERDs (giredestrant, imlunestrant, elacestrant, camizestrant, vepdegestrant, amcenestrant, “oral SERD”), breast cancer (MeSH term “Breast Neoplasms” for PubMed), and randomized trial design. CENTRAL cross-indexes records from MEDLINE, Embase, ClinicalTrials.gov, and the WHO International Clinical Trials Registry Platform. Complete search strategies appear in **Supplementary File S1**. Grey literature sources comprised conference abstract archives of the San Antonio Breast Cancer Symposium (SABCS; 2020–2025), the American Society of Clinical Oncology Annual Meeting (ASCO; 2020–2026), and the European Society for Medical Oncology Congress (ESMO; 2020–2025). ClinicalTrials.gov was searched separately for ongoing and completed trials. We hand-searched reference lists of included studies and two published systematic reviews that incorporated Embase in their search strategies (8, 9), providing independent external validation of search comprehensiveness.

### 2.3 Eligibility criteria

We included Phase II/III randomized controlled trials enrolling adults with ER+/HER2− breast cancer that compared an oral SERD or oral PROteolysis TArgeting Chimera (PROTAC) estrogen receptor degrader, as monotherapy or in combination, against standard endocrine therapy and reported at least one efficacy endpoint. Phase I, single-arm, non-randomized, and preclinical studies were excluded. Conference abstracts presenting Phase II/III results not yet published as full manuscripts were eligible. Per PRISMA 2020 and Cochrane Handbook guidance, conference abstracts constitute grey literature—not unpublished original data—and their inclusion was prespecified in PROSPERO Amendment 1 (May 2026) to minimize publication bias and capture the most current evidence.

### 2.4 Study selection and data extraction

Two investigators independently screened titles and abstracts and assessed full-text articles and conference abstracts per the predefined eligibility criteria. Any disagreements were resolved via group consensus. Data were collected using a standardized, pre-piloted form covering trial characteristics, patient demographics, interventions, comparators, efficacy outcomes, and safety data.

### 2.5 Risk of bias

Risk of bias was assessed with the Cochrane RoB 2 tool (12) across five domains. Phase II trials and conference abstract reports received a default rating of “some concerns,” reflecting open-label designs and incomplete reporting. The full risk of bias assessment for all included trials is detailed in **Supplementary File S3**.

### 2.6 Data synthesis

The primary quantitative synthesis was a random-effects meta-analysis of progression-free survival hazard ratios for oral degrader monotherapy versus endocrine monotherapy in the metastatic setting. Between-study heterogeneity was assessed using *I*² and Cochran’s Q (13); heterogeneity was classified per Cochrane Handbook thresholds (*I*²: 0–30%, low; 30–60%, moderate; 60–100%, substantial) (13). The DerSimonian-Laird estimator was used for τ² in the primary analysis. As a sensitivity analysis, we also applied the restricted maximum likelihood (REML) estimator.

The single adjuvant trial (lidERA) was described narratively. *ESR1*-mutated subgroup analyses pooled hazard ratios in patients with detectable *ESR1* mutations. Combination strategies (evERA, AMEERA-5) and the switching-before-progression design of SERENA-6 were reported narratively, as their designs differed substantially from monotherapy trials. Publication bias was examined via Egger’s regression test and funnel plot inspection. All analyses were performed using the metafor (v5.0-1) and bayesmeta packages in R, with statistical significance set at two-sided α = 0.05. Complete input data (log hazard ratios, standard errors, and source citations) are tabulated in **Supplementary File S5** to facilitate independent reproducibility.

## 3 Results

### 3.1 Study selection

**Figure 1:**
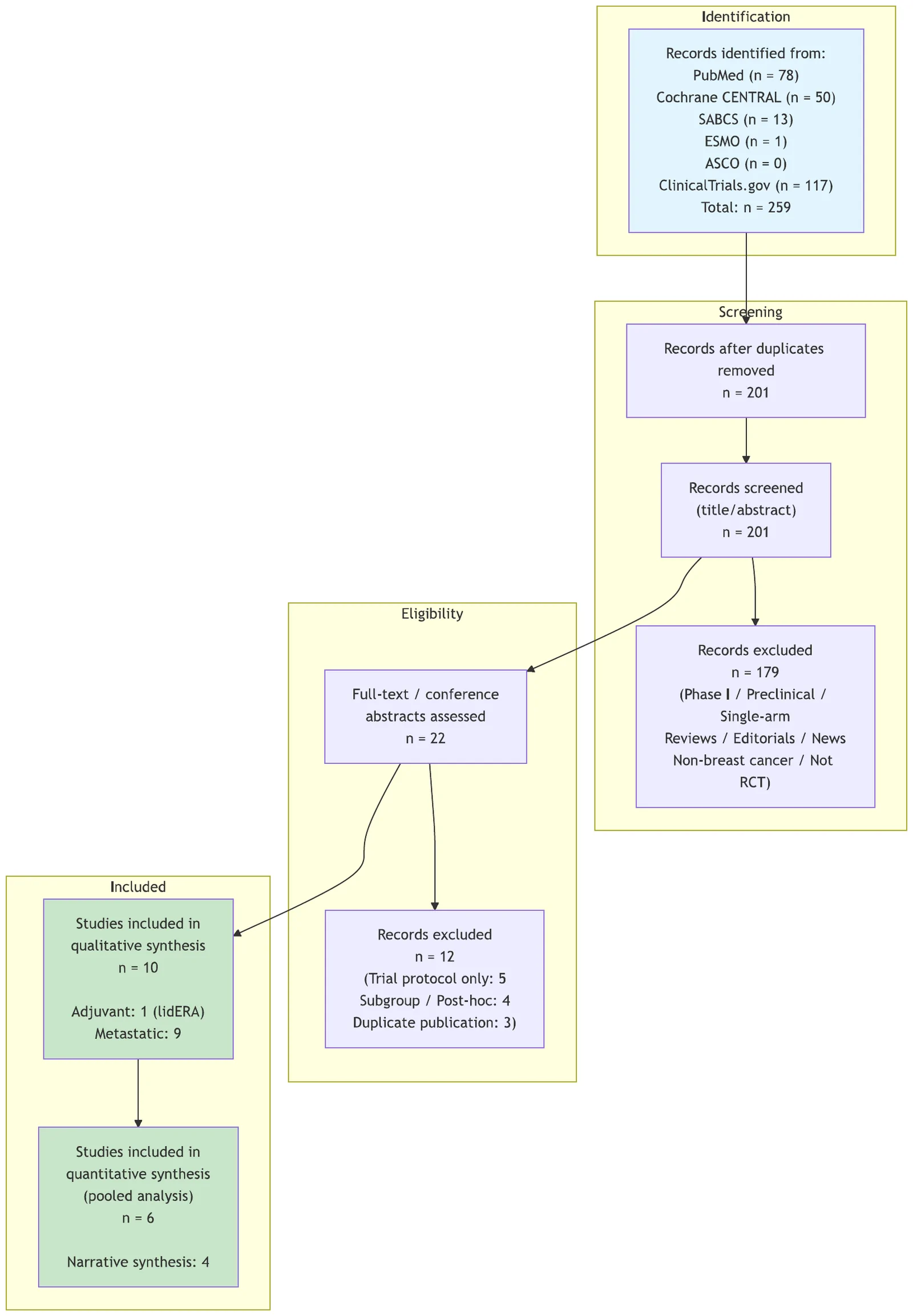
PRISMA 2020 flow diagram.

The systematic search identified 78 records from PubMed and 50 from CENTRAL. The CENTRAL search retrieved no unique eligible randomized controlled trials beyond those already indexed in PubMed. This is attributable to the specificity of the search domain—oral SERD randomized trials in breast cancer represent a narrow, well-circumscribed literature concentrated in major oncology and general medical journals, all of which are comprehensively indexed in MEDLINE. The 50 CENTRAL records comprised PubMed-indexed trial reports, ClinicalTrials.gov registry entries, and conference proceedings already captured through our dedicated grey literature search of SABCS, ESMO, and ASCO meeting abstracts. Thirteen records were identified from SABCS, 1 from ESMO, 0 from ASCO, and 117 from ClinicalTrials.gov (**Figure 1**). After duplicate removal and title/abstract screening, 22 full-text articles and conference abstracts were assessed. Ten randomized controlled trials met inclusion criteria: one in the adjuvant setting and nine in the metastatic setting. Six metastatic monotherapy trials were included in the primary pooled analysis; three (SERENA-6, evERA, AMEERA-5) employed combination or unique designs and were synthesized narratively. The PRISMA 2020 checklist is presented in **Supplementary File S2**. A list of excluded studies with reasons for exclusion is provided in **Supplementary File S4**. **Table 1** provides a detailed PRISMA study selection summary across all information sources.

**Table 1:** Study Selection Summary (for PRISMA).

Source	Records identified	After duplicate removal	Included
PubMed	78	—	10§
Cochrane CENTRAL	50	—	0
SABCS 2020–2025	13	—	1
ESMO 2020–2025	1	—	1
ASCO 2020–2026	0	—	0
ClinicalTrials.gov	117	—	0¥
**Total**	**259**	**[TBD]**	**10**

• § Includes all 10 primary RCT reports; 8 as full-text manuscripts, 2 as conference abstracts.

• ¥ No additional trials meeting eligibility criteria beyond those already captured via PubMed and conference abstract searches. Ongoing trials (EMBER-4, CAMBRIA-2, ELEGANT, persevERA, SERENA-4) are described narratively.Bold values indicate primary pooled analysis results.

### 3.2 Study characteristics

The ten included trials enrolled 9,114 patients between 2020 and 2025 (**Table 2**). Four metastatic trials were Phase III (EMERALD, EMBER-3, VERITAC-2, AMEERA-5) and five were Phase II (SERENA-2, acelERA, AMEERA-3, and two additional early-phase randomized studies). Prior CDK4/6 inhibitor exposure ranged from 42% to 100%. ESR1 mutation prevalence in the evaluable populations ranged from 39% to 55%. Median follow-up ranged from 7.9 months (acelERA) to 32.4 months (lidERA). All trials were open-label except AMEERA-5 (double-blind, double-dummy). Progression-free survival was the primary endpoint in the metastatic setting; invasive disease-free survival in the adjuvant setting.

**Table 2:** Characteristics of Included Randomized Controlled Trials.

Trial	Drug	Phase	Setting	N	Prior CDK4/6i (%)	ESR1-mut (%)	Comparator	Primary endpoint	Median FU (mo)	Data source
Adjuvant setting										
lidERA (NCT04961996)	Giredestrant 30 mg	III	Adjuvant early BC (stage I–III)	4,170	N/A (adjuvant)	NR	Tamoxifen or AI (SoC ET)	iDFS	32.4	SABCS 2025 GS1–10 [abstract]
Metastatic setting										
EMERALD (NCT03778931)	Elacestrant 400 mg	III	mBC ≥2nd line	477	100†	47.8	Fulvestrant/AI/exemestane (SoC)	PFS (BICR)	NR	J Clin Oncol 2022 [full]
EMBER-3 (NCT04975308)	Imlunestrant ± abemaciclib	III	mBC ≥2nd line	874	100	29.3	Fulvestrant or exemestane	PFS	28.5*	N Engl J Med 2025; Ann Oncol 2026 [full]
SERENA-2 (NCT04214288)	Camizestrant 75/150 mg	II	mBC ≥2nd line	240	~70	NR	Fulvestrant 500 mg	PFS (INV)	16.6	Lancet Oncol 2024 [full]
SERENA-6 (NCT04964934)	Camizestrant 75 mg + CDK4/6i	III	1L mBC, ESR1-mut by ctDNA	315	100#	100#	AI + CDK4/6i (continue)	PFS (INV)	12.6	N Engl J Med 2025 [full]
acelERA (NCT04576455)	Giredestrant 30 mg	II	mBC 2–3rd line	303	42	38.8	Fulvestrant (75%) or AI (25%)	PFS (INV)	7.9	J Clin Oncol 2024 [full]
AMEERA-3 (NCT04059484)	Amcenestrant 400 mg	II	mBC ≥2nd line	290	79	42.6	Fulvestrant/AI/tamoxifen (TPC)	PFS (ICR)	11.2	J Clin Oncol 2023 [full]
AMEERA-5 (NCT04478266)	Amcenestrant + palbociclib	III	1L mBC	1,068	0	NR	Letrozole + palbociclib	PFS (INV)	8.4	J Clin Oncol 2024 [full]
VERITAC-2 (NCT05654623)	Vepdegestrant 200 mg	III	mBC post-CDK4/6i	624	100†	43.3	Fulvestrant 500 mg	PFS (BICR)	NR	N Engl J Med 2025 [full]
evERA (NCT05306340)	Giredestrant + everolimus	III	mBC post-CDK4/6i	373	100	55	SoC ET + everolimus	PFS	18.6	ESMO 2025 LBA16 [abstract]

AI, aromatase inhibitor; BICR, blinded independent central review; BC, breast cancer; CDK4/6i, cyclin-dependent kinase 4/6 inhibitor; ctDNA, circulating tumor DNA; ESR1-mut, ESR1 mutation positive; ET, endocrine therapy; FU, follow-up; iDFS, invasive disease-free survival; ICR, independent central review; INV, investigator-assessed; mBC, metastatic breast cancer; NR, not reported; PFS, progression-free survival; SoC, standard of care; TPC, treatment of physician’s choice.

† Mandatory prior CDK4/6 inhibitor therapy per trial eligibility criteria. - * 28.5 months for the updated overall survival analysis (Ann Oncol 2026); primary PFS analysis had shorter follow-up. - # SERENA-6 enrolled exclusively patients with emerging ESR1 mutations detected by ctDNA surveillance; all patients were receiving first-line AI + CDK4/6i at enrolment.

### 3.3 Adjuvant setting

**Figure 2:**
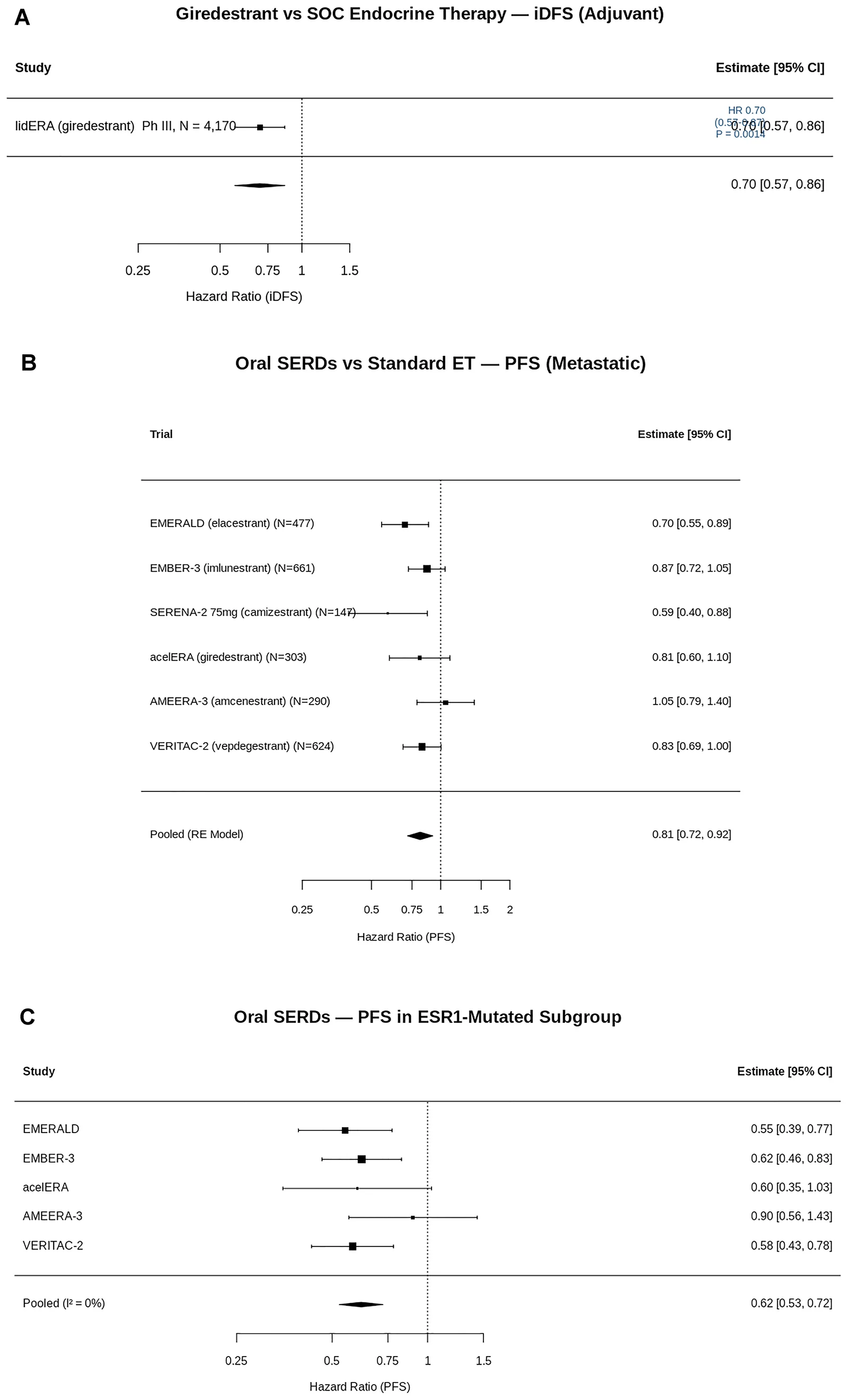
Forest plots. **(A)** Invasive disease-free survival for adjuvant giredestrant versus standard endocrine therapy (lidERA). **(B)** Progression-free survival pooled analysis of oral degrader monotherapy versus endocrine monotherapy in the metastatic setting. **(C)** Progression-free survival in the ESR1-mutated subpopulation.

The lidERA trial (NCT04961996; giredestrant 30 mg daily vs. standard-of-care endocrine therapy; n = 4,170) significantly improved invasive disease-free survival (HR 0.70; 95% CI 0.57–0.87; *P* = 0.0014; **Figure 2A**) at a median follow-up of 32.4 months. Three-year invasive disease-free survival was 92.4% with giredestrant and 89.6% with standard therapy (absolute improvement 2.8%). The distant recurrence-free interval similarly favored giredestrant (HR 0.69; 95% CI 0.54–0.89). Overall survival data were immature (HR 0.79; 95% CI 0.56–1.12; P = 0.19). Benefit was consistent across comparators (aromatase inhibitor: HR 0.73; tamoxifen: HR 0.53). Treatment discontinuation due to adverse events was lower with giredestrant (5.3% vs. 8.2%).

Three confirmatory adjuvant Phase III trials are ongoing (**Table 3**): EMBER-4 (imlunestrant; n ≈ 8,000; enrollment completed December 2025), CAMBRIA-2 (camizestrant; n = 5,500), and ELEGANT (elacestrant; n = 4,220).

**Table 3:** Ongoing Adjuvant Phase III Oral SERD Randomized Controlled Trials.

Trial	Drug	Sponsor	NCT	Phase	Planned N	Comparator	Primary endpoint	Status	Estimated primary completion
EMBER-4	Imlunestrant	Eli Lilly	NCT05514054	III	~8,000	Tamoxifen or AI (SoC ET)	iDFS	Enrollment completed Dec 2025	Oct 2027
CAMBRIA-2	Camizestrant	AstraZeneca	NCT05952557	III	5,500	Tamoxifen or AI (SoC ET)	IBCFS	Actively recruiting	TBD*
ELEGANT	Elacestrant	Stemline (Menarini)	NCT06492616	III	4,220	Continue SoC ET (switch design)†	IBCFS	Actively recruiting	Aug 2029

AI, aromatase inhibitor; ET, endocrine therapy; IBCFS, invasive breast cancer-free survival; iDFS, invasive disease-free survival; SoC, standard of care.

*CAMBRIA-2 treatment duration up to 7 years with 10-year follow-up; primary completion likely beyond 2030. - †ELEGANT employs a switch design: patients who have completed 24–60 months of adjuvant endocrine therapy are randomized to continue standard ET or switch to elacestrant for the remaining treatment duration (up to 5 additional years).

Rationale for inclusion: These three trials represent the confirmatory adjuvant evidence base that will define the role of oral SERDs in early breast cancer. EMBER-4 and ELEGANT evaluate different oral SERDs; CAMBRIA-2 provides a third agent in a similar population. Together with lidERA (giredestrant), these trials constitute the totality of Phase III adjuvant oral SERD investigation.

Additional Phase III trials (first-line metastatic, beyond scope of adjuvant table): - persevERA (NCT04546009): Giredestrant + palbociclib vs. letrozole + palbociclib, 1L metastatic - SERENA-4 (NCT04711252): Camizestrant + palbociclib vs. anastrozole + palbociclib, 1L metastatic

### 3.4 Metastatic primary meta-analysis

Six monotherapy trials (n = 2,502) contributed progression-free survival data (EMERALD, EMBER-3, SERENA-2–75 mg (14), acelERA (15), AMEERA-3 (16), VERITAC-2 (17)). Random-effects meta-analysis showed a significant benefit favoring oral degraders (pooled HR 0.815; 95% CI 0.718–0.924; *P* = 0.0014; **Figure 2B**). Heterogeneity across trials was low to moderate (*I*² = 35.2%; τ² = 0.0085; Q = 7.72, df = 5, *P* = 0.17). Individual trial HRs varied from 0.59 (SERENA-2) to 1.05 (AMEERA-3), and five out of six trials favoured oral degraders over control therapy.

### 3.5 ESR1-mutated subgroup

Five trials contributed *ESR1*-mutated progression-free survival data (**Figure 2C**). The pooled hazard ratio was 0.617 (95% CI 0.527–0.723; *P* < 0.0001). Notably, between-study heterogeneity was completely absent (*I*² = 0%; Q = 3.14, df = 4, *P* = 0.54), indicating remarkably consistent treatment effects across different agents and trial designs. Individual hazard ratios ranged from 0.55 (EMERALD) to 0.90 (AMEERA-3), all below 1.0. In *ESR1* wild-type tumors, by contrast, the pooled effect was attenuated and not statistically significant (HR 0.91; 95% CI 0.78–1.06; *P* = 0.23; *P* for interaction = 0.001).

### 3.6 Sensitivity analyses

**Figure 3:**
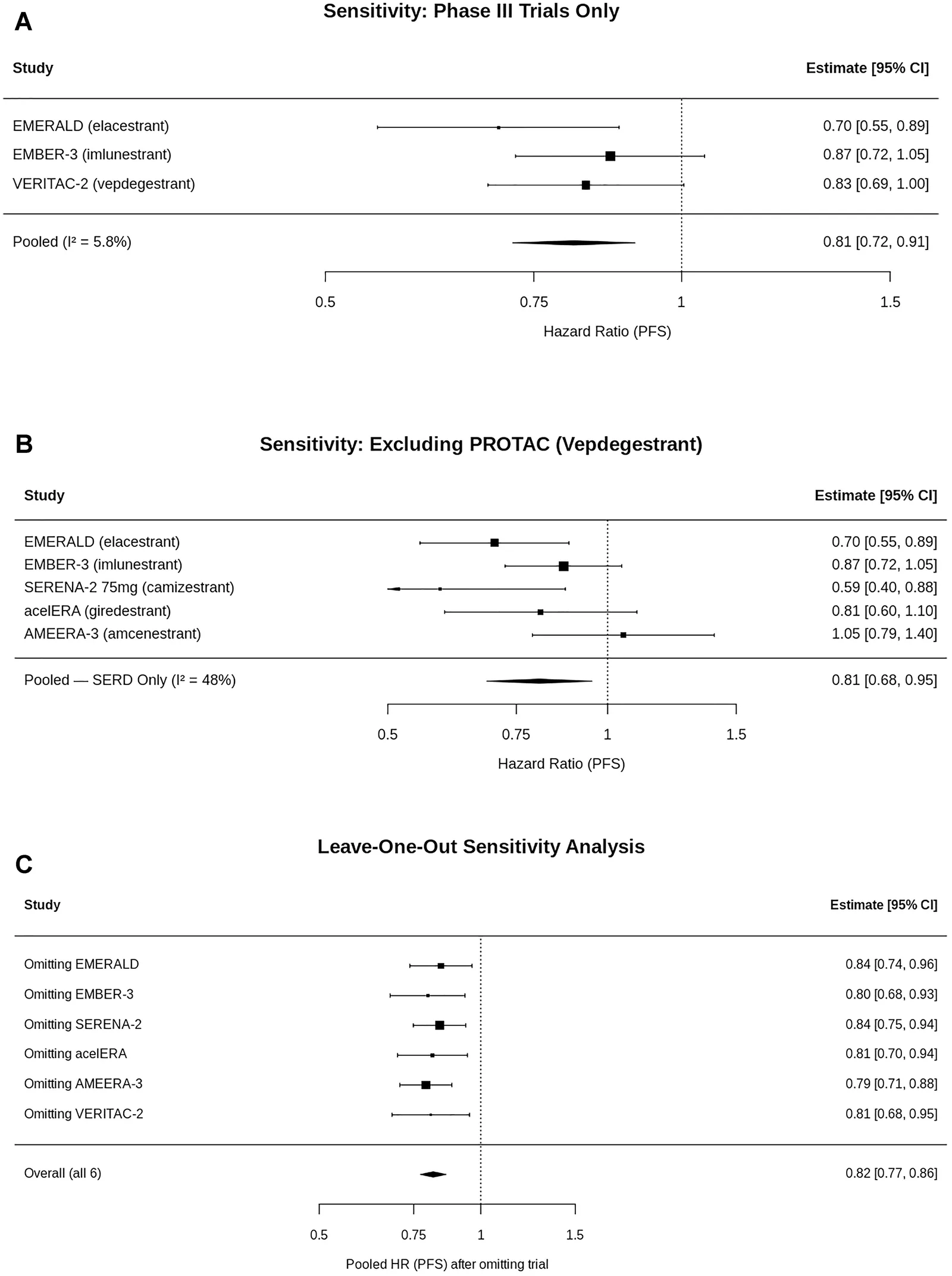
Sensitivity analyses: **(A)** Restriction to Phase III trials only (EMERALD, EMBER-3, VERITAC-2; *I*² = 5.8%). **(B)** Exclusion of PROTAC agent vepdegestrant — SERD-only analysis (*I*² = 48.0%). **(C)** Leave-one-out analysis.

**Figure 4:**
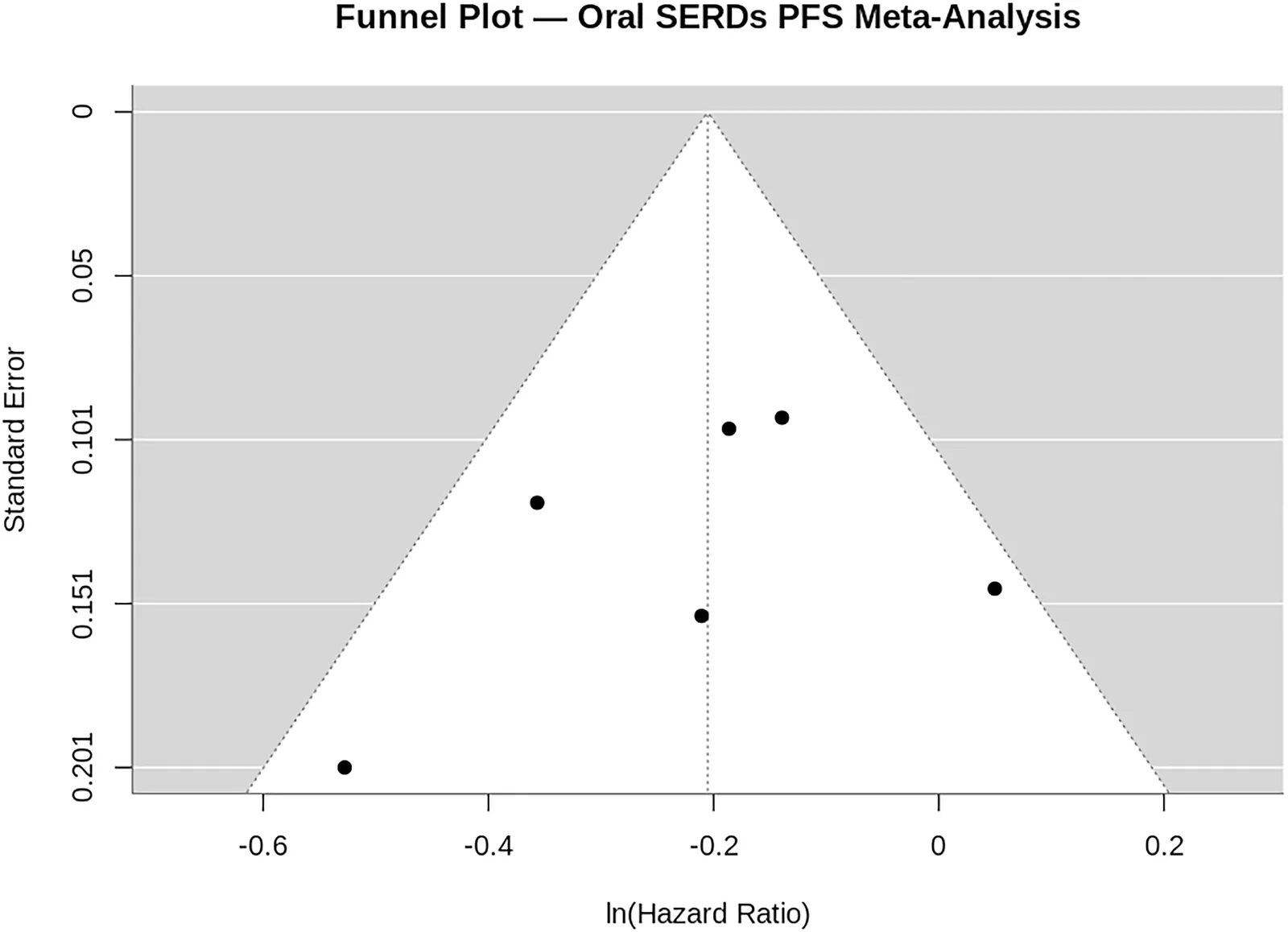
Funnel plot for assessment of publication bias.

The primary finding was robust across all prespecified sensitivity analyses (**Table 4**; **Figure 3**). Use of the restricted maximum likelihood (REML) estimator for τ² yielded a comparable pooled estimate (HR 0.817, 95% CI 0.729–0.915), confirming that the choice of heterogeneity variance estimator did not influence conclusions. Restriction to Phase III trials only (EMERALD, EMBER-3, VERITAC-2) yielded a nearly identical estimate (HR 0.811; 95% CI 0.720–0.913; *I*² = 5.8%; **Figure 3A**). Exclusion of the PROTAC agent vepdegestrant (17) did not materially alter the result (HR 0.807; 95% CI 0.683–0.952; *I*² = 48.0%; **Figure 3B**). Leave-one-out pooled estimates ranged from 0.790 to 0.843 (**Figure 3C**). Bayesian re-analysis with a Jeffreys prior yielded a posterior median hazard ratio of 0.814 (95% credible interval 0.678–0.969). Egger’s regression test did not detect statistically significant funnel plot asymmetry (intercept = 0.008; *P* = 0.40; **Figure 4**). Given that only six trials were included, statistical power for detecting publication bias is limited, and this result should be interpreted as exploratory.

**Table 4:** Meta-Analysis Results — Progression-Free Survival **(A)**. Overall Progression-Free Survival (Monotherapy vs. Endocrine Therapy).

Trial	Phase	N	HR	95% CI	Weight (%)	P
A. Overall progression-free survival (monotherapy vs. endocrine therapy).						
EMERALD (elacestrant)	III	477	0.70	0.55–0.88	27.8	0.002
EMBER-3 (imlunestrant)	III	661	0.87	0.72–1.04	18.9	0.12
SERENA-2 75mg (camizestrant)	II	147	0.59	0.40–0.88*	9.4	0.017
acelERA (giredestrant)	II	303	0.81	0.60–1.10	10.6	0.18
AMEERA-3 (amcenestrant)	II	290	1.05	0.79–1.40	7.7	0.64
VERITAC-2 (vepdegestrant)	III	624	0.83	0.69–1.01	25.7	0.07
**Pooled (Random Effects)**	—	**2,502**	**0.815**	**0.718–0.924**	**100**	**0.0014**
* 90% CI (0.42–0.82) from original publication converted to 95% CI via SE back-calculation for meta-analytic pooling. **Between-study heterogeneity: I² = 35.2% (moderate); τ² = 0.0085; Cochran’s Q = 7.72; df = 5; P = 0.17.**						
B. ESR1-mutated subgroup (monotherapy vs. endocrine therapy)						
Trial	HR		95% CI		n (ESR1-mut)	
EMERALD (elacestrant)	0.55		0.39–0.77		228	
EMBER-3 (imlunestrant)	0.62		0.46–0.82		256	
acelERA (giredestrant)	0.60		0.35–1.03		90	
AMEERA-3 (amcenestrant)	0.90		0.57–1.44		~124	
VERITAC-2 (vepdegestrant)	0.58		0.43–0.78		270	
**Pooled (Random Effects)**	**0.617**		**0.527–0.723**		**968**	
**Between-study heterogeneity: I² = 0% (none); τ² = 0; Cochran’s Q = 3.14; df = 4; P = 0.54.** **P for interaction (ESR1-mut vs. ESR1-wt): 0.001.**						
C. Sensitivity analyses						
Analysis		k	Pooled HR	95% CI	I² (%)	τ²
**Primary analysis** (all 6 trials)		6	0.815	0.718–0.924	35.2	0.0085
**Phase III only** (EMERALD, EMBER-3, VERITAC-2)		3	0.811	0.720–0.913	5.8	0.0009
**Excluding PROTAC** (SERD only)		5	0.807	0.683–0.952	48.0	0.0166
Leave-one-out range		5†	0.790–0.843	—	—	—
**REML estimator** (sensitivity)		6	0.817	0.729–0.915	21.7	—
**Bayesian** (Jeffreys prior)		6	0.814‡	0.678–0.969§	—	—
**• † Each value represents the pooled HR after exclusion of one trial at a time (range across all iterations).** **• ‡ Posterior median hazard ratio.** **• § 95% credible interval.**						
D. Publication bias						
Test			Result		Interpretation	
Egger’s regression test			Intercept = 0.008; P = 0.397		Exploratory; no significant asymmetry detected (k = 6, power limited)	
Funnel plot			Symmetric on visual inspection		Exploratory assessment only	
E. Trial-level safety summary						
Trial		Drug	N (SERD arm)	G≥3 TEAE (%)	Discontinuation due to AE (%)	Notable Toxicity
lidERA		Giredestrant	2,084	NR	5.3 vs. 8.2 (standard ET)	Arthralgia 48%, hot flush 27%
EMERALD		Elacestrant	239	7.2 vs. 3.1	3.4 vs. 0.9	Nausea 35% (any grade)
EMBER-3		Imlunestrant	331	17.1 vs. 20.7	NR	Fatigue, diarrhea
SERENA-2		Camizestrant 75mg	74	No G≥3 TRAE in >2 pts	NR	Photopsia 12%, bradycardia 5%
acelERA		Giredestrant	151	17.3 vs. 11.8	1.3 vs. 2.0	AST↑ 15%, arthralgia 12%
AMEERA-3		Amcenestrant	143	21.7 vs. 15.6	3.5 vs. 1.4	Nausea 20%
VERITAC-2		Vepdegestrant	313	23.4 vs. 17.6	2.9 vs. 0.7	Comparable to fulvestrant
evERA		Gired + everolimus	~187	NR	17 vs. 12	Stomatitis 47% (mTORi)

TEAE , treatment-emergent adverse event; TRAE , treatment-related adverse event; NR , not reported.Bold values indicate the pooled(meta-analysis) estimate.

### 3.7 Combination strategies and unique designs

SERENA-6 (NCT04964934; n = 315) (20) employed a switching-before-progression design in first-line patients with emerging *ESR1* mutations detected by ctDNA surveillance. Camizestrant significantly prolonged progression-free survival (median 16.0 vs. 9.2 months; HR 0.44; 95% CI 0.31–0.60) and delayed quality-of-life deterioration (HR 0.54; 95% CI 0.34–0.84).

evERA (18) (NCT05306340; n = 373) evaluated giredestrant plus everolimus versus endocrine therapy plus everolimus in the post-CDK4/6 inhibitor setting, demonstrating significant progression-free survival improvement overall (HR 0.56; 95% CI 0.44–0.71) and in *ESR1*-mutated tumors (HR 0.38; 95% CI 0.27–0.54).

AMEERA-5 (19) (NCT04478266; n = 1,068), comparing amcenestrant plus palbociclib with letrozole plus palbociclib, was stopped for futility (HR 1.21; 95% CI 0.94–1.56). Pharmacokinetic analyses identified amcenestrant as a moderate CYP3A4 inducer reducing palbociclib exposure by approximately 35%.

### 3.8 Safety

Across the six metastatic monotherapy trials, grade ≥3 treatment-emergent adverse events occurred in 17–23% of patients receiving oral degraders and 12–18% of those receiving standard endocrine therapy. Treatment discontinuation due to adverse events was infrequent (1.3–5.3%). Agent-specific toxicities included nausea with elacestrant (35% any grade) and visual effects plus sinus bradycardia with camizestrant (both predominantly grade 1–2, rarely leading to discontinuation). Vepdegestrant exhibited a tolerability profile comparable to fulvestrant (grade ≥3 adverse events 23.4% vs. 17.6%; discontinuation 2.9% vs. 0.7%).

Among combination regimens, the evERA trial of giredestrant plus everolimus demonstrated higher rates of mTOR-inhibitor-class toxicities (stomatitis 47%, diarrhea 27%, anemia 24%) and a higher discontinuation rate (17% vs. 12%). AMEERA-5, which combined amcenestrant with palbociclib, was limited by a pharmacokinetic drug-drug interaction rather than additive toxicity per se.

In the adjuvant lidERA trial, giredestrant exhibited a safety profile comparable to standard-of-care endocrine therapy, with numerically lower treatment discontinuation (5.3% vs. 8.2%) and similar rates of arthralgia (48% vs. 47%) and hot flushes (27% vs. 29%). No new safety signals were identified across any setting.

### 3.9 Risk of bias

The four Phase III metastatic trials with full publication were rated as low risk of bias. The complete Cochrane RoB 2 assessment by domain for all ten included trials is provided in **Supplementary File S3**. Phase II trials and conference abstract reports were rated as having some concerns due to open-label designs and incomplete reporting. The overall risk of bias was considered acceptable for pooled synthesis.

## 4 Discussion

This comprehensive analysis generates three key findings with important clinical implications. First, giredestrant is the first oral SERD—and the first novel endocrine agent in 25 years—to achieve statistically significant improvement in invasive disease-free survival in the adjuvant setting. Second, the updated pooled analysis confirms a consistent progression-free survival benefit across this drug class in metastatic disease (HR 0.815). Third, the *ESR1*-mutated subgroup shows complete homogeneity of treatment effect (*I*² = 0%) across five independent trials, strongly supporting *ESR1* mutation as a predictive biomarker for this drug class.

### 4.1 Adjuvant setting

The lidERA trial represents the most significant development in adjuvant endocrine therapy since the introduction of aromatase inhibitors. The absolute 2.8% improvement in three-year invasive disease-free survival is clinically meaningful in the adjuvant context, where modest absolute differences translate into substantial absolute numbers of prevented recurrences. The consistency of benefit whether the comparator was tamoxifen (HR 0.53) or an aromatase inhibitor (HR 0.73), combined with a more favorable tolerability profile, positions giredestrant as a potential new adjuvant standard of care pending regulatory review.

The adjuvant oral SERD landscape will expand substantially. EMBER-4 (imlunestrant; n ≈ 8,000) completed enrollment in December 2025. CAMBRIA-2 (camizestrant; n = 5,500) and ELEGANT (elacestrant switch strategy; n = 4,220) remain actively enrolling. It should be noted that ongoing adjuvant trials employ different primary endpoints: lidERA and EMBER-4 use invasive disease-free survival (iDFS), whereas CAMBRIA-2 and ELEGANT use invasive breast cancer-free survival (IBCFS). While these endpoints are similar, IBCFS excludes second primary non-breast cancers and deaths from non-breast cancer causes, which may yield slightly higher event-free rates compared with iDFS. This difference warrants attention when interpreting future cross-trial comparisons. Systematic mapping of these trials, as provided in this review, establishes a reference framework for the expected evolution of the adjuvant endocrine therapy paradigm.

### 4.2 Metastatic setting and comparison with prior systematic reviews

Our pooled hazard ratio of 0.815 extends prior syntheses (8, 9) by incorporating two trials (VERITAC-2, evERA) that post-date earlier literature searches. Kirmani and colleagues (8) reported a pooled hazard ratio of 0.80 (95% CI 0.62–1.04; *P* = 0.09) across eight trials, and Rodrigues and colleagues (9) reported 0.79 (95% CI 0.70–0.89) across six trials. The narrower confidence interval and statistical significance of our estimate (*P* = 0.0014) reflect the addition of positive data from VERITAC-2 and the updated evidence base. Prior narrative reviews have also addressed the oral SERD landscape in advanced breast cancer, including comprehensive discussions of resistance mechanisms and clinical trial design considerations (21, 22).

To contextualize the rapidly evolving and potentially redundant literature in this space, **Table 5** provides a trial-by-trial comparison of inclusion across the three most recent systematic reviews. Our review uniquely includes the adjuvant lidERA trial and the evERA combination trial, both of which were unavailable at the time of the prior searches.

**Table 5:** Cross-Comparison of Trial Inclusion Across Recent Oral SERD Systematic Reviews.

Trial	Drug	Setting	Phase	N	Kirmani 2026 (8)	Rodrigues 2026 (9)	Present Review
EMERALD	Elacestrant	Metastatic	III	477	✓	✓	✓
EMBER-3	Imlunestrant	Metastatic	III	874	✓	✓	✓
SERENA-2	Camizestrant	Metastatic	II	240	✓	✓	✓
acelERA	Giredestrant	Metastatic	II	303	✓	—	✓
AMEERA-3	Amcenestrant	Metastatic	II	290	✓	✓	✓
AMEERA-5	Amcenestrant + palbociclib	Metastatic	III	1,068	✓	—	✓ (narrative)
VERITAC-2	Vepdegestrant (PROTAC)	Metastatic	III	624	—	—	✓
SERENA-6	Camizestrant	Metastatic	III	315	—	✓	✓ (narrative)
evERA	Giredestrant + everolimus	Metastatic	III	373	—	—	✓ (narrative)
lidERA	Giredestrant	Adjuvant	III	4,170	—	—	✓ (narrative)

• ✓ = included in primary pooled/meta-analysis.

• “narrative” = included in qualitative/narrative synthesis only (combination design, unique trial design, or single-trial setting precluding pooled analysis).

• “—” = trial not included (literature search closure pre-dated trial data availability, or trial not meeting inclusion criteria).

• Kirmani et al. (8): *Cancer Treat Rev* 2026;143:103093. Literature search closed September 2025.

• Rodrigues et al. (9): *Breast Cancer Res Treat* 2026;217:16. Literature search closed ~mid-2025.

Key differentiators of the present review:

• First systematic review to include adjuvant lidERA data (SABCS 2025), which was unavailable at the time of both prior literature searches.

• First to include VERITAC-2 (vepdegestrant, PROTAC) and evERA (giredestrant + everolimus), both reported after prior search closures.

• Only review to include all available randomized evidence, including negative/discontinued trials (AMEERA-3, AMEERA-5), without selective exclusion.

Two analytical decisions merit emphasis. First, we included AMEERA-3—the only trial with an overall hazard ratio nominally above 1.0 (HR 1.05, 95% CI 0.79–1.40)—in the primary analysis. Whether it is appropriate to treat a trial of a subsequently discontinued agent (amcenestrant) as part of a homogeneous drug-class effect warrants scrutiny. The leave-one-out analysis confirmed that its removal shifted the pooled estimate only marginally (0.815 to 0.790), suggesting that its inclusion does not distort the class-level conclusion. Notably, the *ESR1*-mutated subpopulation in the same trial showed a numerically favorable HR of 0.90, consistent with the class-wide pattern. Second, the PROTAC agent vepdegestrant (HR 0.83) produced an estimate concordant with the SERD class effect; the increase in heterogeneity upon its exclusion (from *I*² = 35.2% to 48.0%) may reflect the reduced number of studies (k = 5) rather than a mechanistic convergence, and caution is warranted given the wide uncertainty around *I*² at small k.

### 4.3 ESR1 as a predictive biomarker

The consistent therapeutic effect of oral degraders in *ESR1*-mutated tumors (*I*² = 0%) is the most clinically actionable finding of this study. The consistency of the pooled estimate across five trials testing four distinct molecules provides high confidence (*P* < 0.0001 for the pooled HR) that patients with *ESR1*-mutant tumors derive clinically meaningful PFS benefit from these agents. The biological basis is compelling: *ESR1* mutations confer ligand-independent receptor activation that is insensitive to estrogen deprivation by aromatase inhibitors but remains susceptible to direct receptor degradation. This finding reinforces current ASCO and ESMO guideline recommendations for routine *ESR1* mutation testing in patients with advanced breast cancer progressing on prior endocrine therapy (23, 24).

### 4.4 Progression-free survival as the primary endpoint

The conclusions of this review rest primarily on improvement in progression-free survival in the metastatic setting and invasive disease-free survival in the adjuvant setting. Overall survival data were immature across virtually all included trials, precluding meaningful pooled analysis. While PFS is an accepted regulatory endpoint for randomized trials in advanced breast cancer, its surrogacy for overall survival in the context of oral SERD therapy has not been formally validated. Extended follow-up from EMERALD, EMBER-3 (25), and ultimately lidERA will be necessary to confirm whether the PFS and iDFS benefits reported here translate into survival gains. Until mature OS data become available, the clinical benefit of oral SERDs should be interpreted as a meaningful delay in disease progression rather than a proven improvement in survival duration.

### 4.5 Strengths and limitations

The strengths of this review include its prospective PROSPERO registration, the comprehensive search strategy incorporating grey literature, the inclusion of all available randomized evidence without selective exclusion, the prespecified sensitivity analyses spanning frequentist and Bayesian frameworks, and the novel integration of adjuvant and metastatic evidence. Institutional access to Embase, Web of Science, and Scopus was unavailable. This limitation was mitigated through three complementary strategies: (a) CENTRAL cross-indexing of Embase records, which retrieved 50 records—none representing unique eligible trials; (b) independent external validation against two published systematic reviews that included Embase in their search strategies and identified the same set of eligible trials (8, 9); and (c) dedicated hand-searching of SABCS, ESMO, and ASCO meeting abstracts to capture conference-level evidence not yet indexed in bibliographic databases.

Several limitations warrant acknowledgment. The lidERA and evERA data are currently available only as conference abstracts; full manuscripts are pending. Conference abstract inclusion was prespecified in PROSPERO Amendment 1 (May 2026) to capture the most current evidence and prevent publication bias toward older, fully published data. Data for lidERA were extracted from SABCS 2025 Abstract GS1–10 and the accompanying presentation slides. Data for evERA were extracted from ESMO 2025 Abstract LBA16. We acknowledge that extended safety data, complete subgroup analyses, and mature overall survival results will require updating upon full publication.

The metastatic pooled analysis is limited to six trials (k = 6), which constrains statistical power for exploring heterogeneity sources and limits the reliability of publication bias assessment. Furthermore, the meta-analysis focused exclusively on progression-free survival. Other clinically meaningful endpoints—overall survival, duration of response, and quality of life—were not quantitatively evaluated, largely because overall survival data were immature across most included trials and patient-reported outcomes were not uniformly reported. It is noteworthy, however, that SERENA-6 demonstrated a delay in quality-of-life deterioration (HR 0.54), suggesting that progression-free survival gains may translate into symptomatic benefit in selected settings.

Considerable clinical heterogeneity exists across the included trials with respect to the proportion of patients with prior CDK4/6 inhibitor exposure (42–100%), the number and type of prior endocrine therapy lines, and the specific comparator agent (fulvestrant versus aromatase inhibitor versus physician’s choice). These differences reflect genuine variation in clinical practice patterns and trial design rather than methodological flaws. We addressed this heterogeneity through random-effects modeling and prespecified sensitivity analyses (Phase III only, exclusion of PROTAC agent, leave-one-out iteration), which consistently supported the primary pooled estimate. Meta-regression or additional subgroup analyses to formally explore these sources of heterogeneity were precluded by the limited number of trials (k = 6).

The *ESR1* mutation testing methodology, circulating tumor DNA assay platform, and mutation panel content varied across trials (FoundationOne Liquid CDx, Guardant360, PredicineCARE). While the *I*² = 0% finding indicates statistical homogeneity, this does not fully address potential misclassification bias arising from heterogeneous assay platforms with differing analytical sensitivity, limits of detection, and variant allele frequency reporting thresholds. Beyond these technical considerations, the routine implementation of *ESR1*-guided therapy faces practical barriers including the need for standardized, reimbursable ctDNA testing, consensus on optimal testing timing (at progression versus earlier surveillance), and education of community oncologists on assay interpretation. The clinical application of *ESR1*-guided therapy therefore requires both cross-platform validation studies and health-system-level strategies to ensure equitable access to biomarker-driven care.

Clinical heterogeneity exists with respect to lines of prior therapy, comparator type (fulvestrant versus aromatase inhibitor versus physician’s choice), and prior CDK4/6 inhibitor exposure, though random-effects modeling and prespecified subgroup analyses partially address this limitation.

## 5 Conclusions

Oral selective estrogen receptor degraders confer a consistent progression-free survival benefit in advanced ER+/HER2−negative breast cancer, with the most robust and consistent effect in ESR1-mutated tumors (pooled HR 0.617; *I*² = 0%). Giredestrant is the first oral SERD to achieve significant improvement in invasive disease-free survival in the adjuvant setting (HR 0.70), marking a potential new standard of care. This analysis across early and advanced breast cancer may aid clinical decision-making as oral SERDs are increasingly used throughout the disease continuum.

## Data Availability

The original contributions presented in the study are included in the article/**Supplementary Material**. Further inquiries can be directed to the corresponding author.

## Supplementary material

Supplementary File S1 Complete search strategies for PubMed and CENTRAL, including grey literature sources (SABCS, ESMO, ASCO, ClinicalTrials.gov) and external validation details.

Supplementary File S2 PRISMA 2020 checklist with section locations for all 27 reporting items.

Supplementary File S3 Cochrane Risk of Bias 2 (RoB 2) assessments by domain for all ten included randomized controlled trials.

Supplementary File S4 List of studies excluded after full-text or conference abstract review with reasons for exclusion, including competing systematic reviews.

Supplementary File S5 Input data for reproducibility — tabulated hazard ratios, log-transformed effect sizes, standard errors, and R code for all meta-analyses.
