# Supplementary material for: Oral selective estrogen receptor degraders in ER+/HER2− breast cancer: a systematic review with pooled analysis of metastatic randomized trials and narrative synthesis of adjuvant evidence
Source: Front Oncol. 2026 Sep 3;16:1896847. doi: 10.3389/fonc.2026.1896847 (PMC13581930; doi:10.3389/fonc.2026.1896847)
Supplement: Supplementary file 1 [file Table1.docx]

# Supplementary File 1 — Complete PubMed Search Strategy

**Database:** PubMed (MEDLINE)
**Date searched:** April 28, 2026
**Date range:** January 1, 2018 – April 28, 2026
**Records retrieved:** 78

```
#1  (giredestrant[tiab] OR imlunestrant[tiab] OR elacestrant[tiab]
     OR camizestrant[tiab] OR vepdegestrant[tiab] OR amcenestrant[tiab]
     OR "oral SERD"[tiab] OR "oral selective estrogen receptor degrader"[tiab])

#2  ("Breast Neoplasms"[Mesh] OR "breast cancer"[tiab]
     OR "breast carcinoma"[tiab])

#3  ("randomized controlled trial"[Publication Type] OR randomized[tiab]
     OR "phase 2"[tiab] OR "phase 3"[tiab] OR "phase II"[tiab]
     OR "phase III"[tiab])

#4  #1 AND #2 AND #3

Date filter: 2018/01/01 to 2026/04/28
```

## Grey Literature Search

| Source | URL / Access Method | Search Terms | Date Searched | Records |
| --- | --- | --- | --- | --- |
| SABCS | sabcs.org → Past Meetings 2020–2025 | giredestrant OR imlunestrant OR elacestrant OR camizestrant OR vepdegestrant OR amcenestrant OR “oral SERD” | May 2026 | 13 |
| ESMO | oncologypro.esmo.org / Annals of Oncology supplements 2020–2025 | giredestrant imlunestrant elacestrant camizestrant vepdegestrant amcenestrant | May 2026 | 1 |
| ASCO | meetings.asco.org 2020–2026 | giredestrant OR imlunestrant OR elacestrant OR camizestrant OR vepdegestrant OR amcenestrant | May 2026 | 0 |
| ClinicalTrials.gov | clinicaltrials.gov | giredestrant OR imlunestrant OR elacestrant OR camizestrant OR vepdegestrant OR amcenestrant OR “oral SERD” | May 2026 | 117 |

**Total records identified:** 209
**Duplicates removed:** [enter number after deduplication]
**Records screened:** [enter number]
**Full-text/abstract assessed:** 22
**Studies included:** 10
