# Supplementary material for: Oral selective estrogen receptor degraders in ER+/HER2− breast cancer: a systematic review with pooled analysis of metastatic randomized trials and narrative synthesis of adjuvant evidence
Source: Front Oncol. 2026 Sep 3;16:1896847. doi: 10.3389/fonc.2026.1896847 (PMC13581930; doi:10.3389/fonc.2026.1896847)
Supplement: Supplementary file 2 [file Table2.docx]

# Supplementary File 2 — PRISMA 2020 Checklist

| Section | Item | PRISMA 2020 Checklist Item | Location |
| --- | --- | --- | --- |
| **Title** | 1 | Identify the report as a systematic review | Title page |
| **Abstract** | 2 | Structured summary (Background, Methods, Results, Conclusions, Registration) | Abstract |
| **Rationale** | 3 | Describe the rationale for the review in context of existing knowledge | Introduction ¶1-4 |
| **Objectives** | 4 | Provide an explicit statement of the objectives | Introduction ¶5 |
| **Eligibility criteria** | 5 | Specify inclusion and exclusion criteria | Methods §2.3 |
| **Information sources** | 6 | Specify all databases, registers, grey literature sources | Methods §2.2, Supplementary S1 |
| **Search strategy** | 7 | Present full search strategies for all databases | Supplementary S1 |
| **Selection process** | 8 | Specify methods for screening (number of reviewers, independence) | Methods §2.4 |
| **Data collection process** | 9 | Specify methods for data extraction | Methods §2.4 |
| **Data items** | 10 | List and define all outcomes for which data were sought | Methods §2.3 (Outcomes) |
| **Risk of bias** | 11 | Specify methods used to assess risk of bias | Methods §2.5 |
| **Effect measures** | 12 | Specify effect measures (HR, 95% CI) used in synthesis | Methods §2.6 |
| **Synthesis methods** | 13 | Describe methods for data synthesis, handling heterogeneity, sensitivity analyses | Methods §2.6 |
| **Reporting bias** | 14 | Describe methods for assessing publication bias | Methods §2.6 (Egger’s, funnel) |
| **Certainty** | 15 | Describe methods for assessing certainty of evidence | Methods §2.5 (RoB 2) |
| **Study selection** | 16 | Describe results of search and selection process (flow diagram) | Results §3.1, Figure 1 |
| **Study characteristics** | 17 | Present characteristics of each included study | Results §3.2, Table 1 |
| **Risk of bias** | 18 | Present assessments of risk of bias for each included study | Results §3.10, Supplementary S3 |
| **Results of individual studies** | 19 | Present summary statistics for each study | Results §3.5-3.6, Figure 2, Table 2 |
| **Results of syntheses** | 20 | Present results of all meta-analyses, heterogeneity, sensitivity analyses | Results §3.5-3.7, Table 2 |
| **Reporting biases** | 21 | Present assessments of publication bias | Results §3.7, Figure 4 |
| **Certainty of evidence** | 22 | Present assessments of certainty of evidence | Results §3.10 |
| **Discussion** | 23 | Provide general interpretation in context of other evidence | Discussion §4.1-4.4 |
| **Limitations** | 24 | Discuss limitations of the review | Discussion §4.5 |
| **Implications** | 25 | Discuss implications for practice and research | Discussion §4.6, Conclusions |
| **Registration** | 26 | Provide registration information | PROSPERO CRD420261358420 (Methods §2.1, Abstract) |
| **Support** | 27 | Describe sources of funding or other support | [None / to be added] |
| **Competing interests** | 28 | Declare competing interests | [To be added] |
| **Data availability** | 29 | State data sharing availability | [To be added] |
