# Supplementary material for: Oral selective estrogen receptor degraders in ER+/HER2− breast cancer: a systematic review with pooled analysis of metastatic randomized trials and narrative synthesis of adjuvant evidence
Source: Front Oncol. 2026 Sep 3;16:1896847. doi: 10.3389/fonc.2026.1896847 (PMC13581930; doi:10.3389/fonc.2026.1896847)
Supplement: Supplementary file 3 [file Table3.docx]

# Supplementary File 3 — Risk of Bias Assessment (Cochrane RoB 2)

## Summary by Domain

| Trial | Randomization | Deviations | Missing Data | Outcome Measurement | Selective Reporting | **Overall** |
| --- | --- | --- | --- | --- | --- | --- |
| **Phase III — Full Publications** |  |  |  |  |  |  |
| EMERALD (elacestrant) | Low | Some concerns | Low | Low | Low | **Low** |
| EMBER-3 (imlunestrant) | Low | Some concerns | Low | Low | Low | **Low** |
| SERENA-6 (camizestrant) | Low | Low | Low | Low | Low | **Low** |
| VERITAC-2 (vepdegestrant) | Low | Some concerns | Low | Low | Low | **Low** |
| AMEERA-5 (amcenestrant+palbo) | Low | Low | Low | Low | Low | **Low** |
| **Phase II — Full Publications** |  |  |  |  |  |  |
| SERENA-2 (camizestrant) | Some concerns | Some concerns | Low | Some concerns | Low | **Some concerns** |
| acelERA (giredestrant) | Low | Some concerns | Low | Some concerns | Low | **Some concerns** |
| AMEERA-3 (amcenestrant) | Some concerns | Some concerns | Low | Some concerns | Low | **Some concerns** |
| **Conference Abstracts Only** |  |  |  |  |  |  |
| lidERA (giredestrant) | Some concerns | Some concerns | Some concerns | Low | Some concerns | **Some concerns** |
| evERA (giredestrant+evero) | Some concerns | Some concerns | Some concerns | Low | Some concerns | **Some concerns** |

## Domain-Level Justifications

### Randomization Process

- **Low:** Phase III trials with adequate sequence generation and allocation concealment, stratified randomization.
- **Some concerns:** Phase II trials and abstracts without explicit allocation concealment detail. Small Phase II trials (SERENA-2, n=240) with baseline imbalances (acelERA: ESR1-mut 43.6% vs 33.9% in favor of giredestrant arm).

### Deviations from Intended Interventions

- **Low:** Double-blind designs (AMEERA-5 only).
- **Some concerns:** All other trials were open-label, enabling potential differential co-interventions. However, all have standard-of-care comparators with well-characterized safety profiles.

### Missing Outcome Data

- **Low:** All trials maintained high rates of outcome ascertainment. Censoring rates were balanced between arms.

### Outcome Measurement

- **Low:** Phase III trials with BICR (EMERALD, VERITAC-2) or protocol-specified iDFS criteria (lidERA).
- **Some concerns:** Phase II trials and SERENA-6 with investigator-assessed PFS as primary endpoint. acelERA showed notable INV-BIRC PFS discordance (HR 0.81 vs 0.92).

### Selective Reporting

- **Low:** Primary and key secondary endpoints pre-specified on ClinicalTrials.gov and reported in publications.
- **Some concerns:** Conference abstracts (lidERA, evERA) have incomplete reporting of all prespecified outcomes. Full assessment deferred to manuscript publication.

**RoB 2 Tool Reference:** Sterne JAC, Savović J, Page MJ, et al. BMJ. 2019;366:l4898.
