# Supplementary material for: Oral selective estrogen receptor degraders in ER+/HER2− breast cancer: a systematic review with pooled analysis of metastatic randomized trials and narrative synthesis of adjuvant evidence
Source: Front Oncol. 2026 Sep 3;16:1896847. doi: 10.3389/fonc.2026.1896847 (PMC13581930; doi:10.3389/fonc.2026.1896847)
Supplement: Supplementary file 4 [file Table4.docx]

# Supplementary File 4 — Excluded Studies with Reasons

## Studies Excluded After Full-Text/Abstract Review

| # | Citation / PMID | Reason for Exclusion |
| --- | --- | --- |
| 1 | coopERA BC (PMID 37657462) | **Setting mismatch.** Neoadjuvant trial; primary endpoint pathological complete response (pCR) — not iDFS or PFS. Different patient population and treatment context. |
| 2 | EMBER-3 updated OS (PMID 41391667) | **Duplicate.** Updated analysis of EMBER-3 (PMID 39660834 already included). Retained for supplementary OS data extraction only. |
| 3 | EMERALD subgroup analysis (PMID 39087959) | **Subgroup/post-hoc analysis.** Not the primary RCT report. Data extracted from primary paper (PMID 35584336). |
| 4 | SERENA-1 (PMID 38729567) | **Phase I dose escalation/expansion.** Non-randomized; not meeting Phase II/III RCT eligibility criterion. |
| 5 | EMERALD protocol (PMID 31426673) | **Trial design/protocol paper.** No efficacy results reported. |
| 6 | VERITAC-2 protocol (PMID 39072356) | **Trial design/protocol paper.** No efficacy results reported. |
| 7 | CAMBRIA-1 & CAMBRIA-2 protocol (PMID 40017004) | **Trial design/protocol paper.** No efficacy results reported. |
| 8 | AMEERA-5 protocol (PMID 35309087) | **Trial design/protocol paper.** No efficacy results reported. |
| 9 | heredERA protocol (PMID 38789924) | **Trial design/protocol paper.** No efficacy results reported. |
| 10 | Phase I imlunestrant (PMID 39241211) | **Phase I trial.** Non-randomized; dose finding. |
| 11 | Imlunestrant in endometrial cancer (PMID 39442371) | **Wrong indication.** Gynecologic cancer, not breast. |
| 12 | Phase I giredestrant + palbociclib (PMID 37921755) | **Phase I dose escalation.** Non-randomized. |
| 13 | Imlunestrant + HER2 therapy EMBER (PMID 41423595) | **Phase Ib.** Non-randomized; HER2+ population. |
| 14 | Preoperative window imlunestrant (PMID 39377773) | **Window-of-opportunity study.** Not treatment-efficacy RCT. |
| 15 | SERENA-6 PRO analysis (PMID 41125211) | **Subgroup/secondary analysis.** Primary results already included (PMID 40454637). |
| 16 | EMERALD genomic analysis (PMID 41201834) | **Exploratory biomarker analysis.** Not primary RCT report. |
| 17 | Elacestrant post-hoc meta-analysis (PMID 39991467) | **Post-hoc analysis.** Not primary RCT report. |
| 18 | EMBER-3 PRO analysis (PMID 41962306) | **Secondary PRO analysis.** Primary results already included. |

## Systematic Reviews Excluded (Category B — Competing Literature, Not Primary Data)

| Citation | Journal | Year | Reason |
| --- | --- | --- | --- |
| Kirmani et al. | Cancer Treat Rev | 2026 | Existing systematic review (metastatic only); used for discussion context and reference list hand-searching |
| Rodrigues et al. | Breast Cancer Res Treat | 2026 | Existing systematic review (metastatic only); used for discussion context |
| PMID 37871699 | Ann Oncol | 2024 | Existing meta-analysis; results superseded by current more complete analysis |
| PMID 39293125 | Cancer Treat Rev | 2025 | Narrative review; no new data |
| PMID 41654790 | BMC Cancer | 2025 | Adjacent topic (post-CDK4/6i); different scope |
| PMID 41385990 | Cancer Treat Rev | 2026 | Network meta-analysis; different methodology |

## Preclinical / Out of Scope Excluded at Title-Abstract

Approximately 40 records excluded as Phase I single-arm studies, preclinical pharmacokinetic/pharmacodynamic studies, medicinal chemistry publications, non-breast cancer studies, erratum notices, narrative reviews, editorials, and news articles. Complete list available on request.
