# Supplementary material for: Oral selective estrogen receptor degraders in ER+/HER2− breast cancer: a systematic review with pooled analysis of metastatic randomized trials and narrative synthesis of adjuvant evidence
Source: Front Oncol. 2026 Sep 3;16:1896847. doi: 10.3389/fonc.2026.1896847 (PMC13581930; doi:10.3389/fonc.2026.1896847)
Supplement: Supplementary file 5 [file Table5.docx]

# Supplementary File S5 — Input Data for Reproducibility

## Progression-Free Survival Hazard Ratios Used in Primary Meta-Analysis

All hazard ratios and 95% confidence intervals were extracted from primary trial publications (full-text or conference abstracts). For each trial, the log hazard ratio (yi) and standard error (sei) were calculated as:

yi = ln(HR)
sei = [ln(upper 95% CI) − ln(lower 95% CI)] / (2 × 1.96)

### A. Overall PFS (6 trials, DerSimonian-Laird random-effects model)

| Trial | HR | 95% CI | ln(HR) | SE | Source |
| --- | --- | --- | --- | --- | --- |
| EMERALD (elacestrant) | 0.70 | 0.55–0.88 | −0.357 | 0.1185 | Bidard et al., J Clin Oncol 2022 [6] |
| EMBER-3 (imlunestrant) | 0.87 | 0.72–1.04 | −0.139 | 0.0926 | Jhaveri et al., N Engl J Med 2025 [7] |
| SERENA-2 75mg (camizestrant) | 0.59* | 0.40–0.88† | −0.528 | 0.2034 | Oliveira et al., Lancet Oncol 2024 [17] |
| acelERA (giredestrant) | 0.81 | 0.60–1.10 | −0.211 | 0.1564 | Bardia et al., J Clin Oncol 2024 [19] |
| AMEERA-3 (amcenestrant) | 1.051 | 0.789–1.400 | 0.050 | 0.1470 | Tolaney et al., J Clin Oncol 2023 [20] |
| VERITAC-2 (vepdegestrant) | 0.83 | 0.69–1.01 | −0.186 | 0.0998 | Campone et al., N Engl J Med 2025 [22] |

* *SERENA-2 originally reported a 90% CI (0.42–0.82). The SE was back-calculated from the 90% CI:*
*SE = [ln(0.82) − ln(0.42)] / (2 × 1.645) = 0.669 / 3.29 = 0.2034*
† *95% CI converted via: exp[ln(0.59) ± 1.96 × 0.2034] = 0.40 to 0.88*

### B. ESR1-Mutated Subgroup PFS (5 trials)

| Trial | HR | 95% CI | ln(HR) | SE | Source |
| --- | --- | --- | --- | --- | --- |
| EMERALD | 0.55 | 0.39–0.77 | −0.598 | 0.1751 | [6] |
| EMBER-3 | 0.62 | 0.46–0.82 | −0.478 | 0.1492 | [7]; FDA prescribing information |
| acelERA | 0.60 | 0.35–1.03 | −0.511 | 0.2767 | [19] |
| AMEERA-3 | 0.90 | 0.565–1.435 | −0.105 | 0.2498 | [20] |
| VERITAC-2 | 0.58 | 0.43–0.78 | −0.545 | 0.1586 | [22] |

### C. Sensitivity Analyses

**Phase III only (k = 3):** EMERALD + EMBER-3 + VERITAC-2
yi = (−0.357, −0.139, −0.186); sei = (0.1185, 0.0926, 0.0998)

**Excluding PROTAC (k = 5):** EMERALD + EMBER-3 + SERENA-2 + acelERA + AMEERA-3
yi = (−0.357, −0.139, −0.528, −0.211, 0.050); sei = (0.1185, 0.0926, 0.2034, 0.1564, 0.1470)

**REML estimator (k = 6):** Same input as primary analysis
yi = (−0.357, −0.139, −0.528, −0.211, 0.050, −0.186); sei = (0.1185, 0.0926, 0.2034, 0.1564, 0.1470, 0.0998)

### D. Software and Code

All analyses performed using R (version ≥4.0) with packages metafor (v5.0-1) and bayesmeta. The complete analysis script is provided in the supplementary material and available from the corresponding author upon request. Key function calls:

```
library(metafor)
yi  <- c(-0.357, -0.139, -0.528, -0.211, 0.050, -0.186)
sei <- c(0.1185, 0.0926, 0.2034, 0.1564, 0.1470, 0.0998)
res <- rma(yi = yi, sei = sei, method = "DL")
exp(res$b)   # Pooled HR
exp(res$ci.lb); exp(res$ci.ub)  # 95% CI
```

### E. Verification of the Pooled Estimate

Running the code in [D] produces:

- Pooled HR: 0.815 (95% CI 0.718–0.924)
- *I*² = 35.2%, τ² = 0.0085, Q = 7.72, df = 5, *P* = 0.17

These results can be independently verified by any reader using the input data tabulated in Section A.
